# Supplementary material for: Modelling Skylarks (Alauda arvensis) to Predict Impacts of Changes in Land Management and Policy: Development and Testing of an Agent-Based Model
Source: PLoS One. 2013 Jun 6;8(6):e65803. doi: 10.1371/journal.pone.0065803 (PMC3675089; doi:10.1371/journal.pone.0065803)
Supplement: Supporting Information S4 — The skylark ODdox as a zipped archive. (ZIP) [file pone.0065803.s004.zip › Skylark_ODdox/struct_low_pri_pair.html]

ALMaSS Skylark ODdox: LowPriPair< ELEMTYPE > Struct Template Reference


ALMaSS Skylark ODdox
 2.0


- Main Page
- Related Pages
- Classes
- Files

- Class List
- Class Index
- Class Hierarchy
- Class Members

Public Types |
Public Member Functions |
Public Attributes

LowPriPair< ELEMTYPE > Struct Template Reference

Used in event handling.
More...

`#include <lowqueue.h>`

List of all members.

|  |  |
| --- | --- |
| Public Types | |
| typedef LowPriPair | size\_type |
| typedef LowPriPair | value\_type |

|  |  |
| --- | --- |
| Public Member Functions | |
|  | LowPriPair () |
|  | LowPriPair (const ELEMTYPE &a\_element, long a\_priority) |
| bool | operator< (const LowPriPair &a\_another\_pri\_pair) const |

|  |  |
| --- | --- |
| Public Attributes | |
| ELEMTYPE | m\_element |
| long | m\_pri |

---

## Detailed Description

### template<class ELEMTYPE> struct LowPriPair< ELEMTYPE >

Used in event handling.

---

## Member Typedef Documentation

template<class ELEMTYPE>

typedef LowPriPair LowPriPair< ELEMTYPE >::size\_type

template<class ELEMTYPE>

typedef LowPriPair LowPriPair< ELEMTYPE >::value\_type

---

## Constructor & Destructor Documentation

template<class ELEMTYPE>

|  |  |  |  |  |
| --- | --- | --- | --- | --- |
| LowPriPair< ELEMTYPE >::LowPriPair | ( |  | ) |  |

inline

{

}

template<class ELEMTYPE>

|  |  |  |  |
| --- | --- | --- | --- |
| LowPriPair< ELEMTYPE >::LowPriPair | ( | const ELEMTYPE & | *a\_element*, |
|  |  | long | *a\_priority* |
|  | ) |  |  |

inline

{

m\_element = a\_element;

m\_pri = a\_priority;

}

---

## Member Function Documentation

template<class ELEMTYPE>

|  |  |  |  |  |  |
| --- | --- | --- | --- | --- | --- |
| bool LowPriPair< ELEMTYPE >::operator< | ( | const LowPriPair< ELEMTYPE > & | *a\_another\_pri\_pair* | ) | const |

inline

References LowPriPair< ELEMTYPE >::m\_pri.

{

return m\_pri > a\_another\_pri\_pair.m\_pri;

}

---

## Member Data Documentation

template<class ELEMTYPE>

ELEMTYPE LowPriPair< ELEMTYPE >::m\_element

Referenced by Farm::HandleEvents(), and Farm::~Farm().

template<class ELEMTYPE>

long LowPriPair< ELEMTYPE >::m\_pri

Referenced by Farm::HandleEvents(), and LowPriPair< ELEMTYPE >::operator<().

---

The documentation for this struct was generated from the following file:

- lowqueue.h


- LowPriPair
- Generated on Thu Jan 10 2013 13:15:36 for ALMaSS Skylark ODdox by
   1.8.1.1
